# Preliminary diagnosis of medial meniscus posterior root tears using the Rosenberg radiographic view

**DOI:** 10.1186/s43019-019-0011-5

**Published:** 2019-09-18

**Authors:** Yuya Kodama, Takayuki Furumatsu, Yusuke Kamatsuki, Takaaki Hiranaka, Tomohiro Takahata, Masayuki Sadakane, Haruhiko Ikuta, Masaharu Yasumitsu, Toshifumi Ozaki

**Affiliations:** 1 Department of Orthopaedic Surgery, Okayama University Graduate School of Medicine, Dentistry, and Pharmaceutical Sciences, 2-5-1 Shikata-cho, Kita-ku, Okayama, 700-8558 Japan; 2 Department of Orthopaedic Surgery, Iwakuni Clinical Center, 1-1-1 Atagomachi, Iwakuni, Yamaguchi, 740-8510 Japan

**Keywords:** Medial meniscus, Posterior root tear, Meniscal extrusion, Joint space, Rosenberg view, Weight-bearing radiograph

## Abstract

**Purpose:**

To verify the effectiveness of detecting medial meniscus posterior root tears (MMPRTs) using weight-bearing posterior-anterior (PA) radiographs.

**Materials and methods:**

Twenty-three patients were diagnosed with an MMPRT using magnetic resonance imaging (Group A), with 23 matched individuals forming the control group (Group B). The distance between medial tibial eminence and the lateral edge of the medial femoral condyle (MTE–MFC distance) and medial joint space (MJS) width were measured on weight-bearing PA radiographs, with the knee flexed at 45° (Rosenberg view). Absolute medial meniscus extrusion (MME) was measured on magnetic resonance images.

**Results:**

The MTE–MFC distance was greater and the MJS width was smaller in Group A than Group B (7.7 ± 1.7 mm versus 6.0 ± 1.24 mm and 3.2 ± 0.8 mm versus 4.5 ± 0.7 mm, respectively; *P* < 0.05). The MTE–MFC distance and MJS width correlated with MME (r = 0.603 and 0.579, respectively; *P* < 0.05), and the extent of MME was greater in Group A than Group B (4.1 ± 1.1 mm versus 1.8 ± 1.5 mm, respectively; *P* < 0.05).

**Conclusions:**

MMPRTs increase the MTE–MFC distance and decrease the MJS width, with these measurements correlating to the MME. Therefore, measurement of the MTE–MFC distance and MJS width on the Rosenberg view could be a useful preliminary method for the diagnosis of an MMPRT.

**Level of evidence:**

IV

## Introduction

Medial meniscus posterior root tears (MMPRTs) disrupt the continuity of the circumferential fibers, leading to loss of hoop tension, loss of load-sharing ability, abnormal rotation of the tibia, and unacceptable peak pressures [1–4]. These pathological characteristics lead to degenerative arthritic changes in the knee joint [1, 3].

The tibial intercondylar eminence can limit inward and outward knee movement, with the vertical axis for knee joint rotation being located in the medial tibial eminence (MTE) [5]. The presence of a tibiofemoral subluxation, evaluated using standing radiographs, is a predisposing factor for medial knee joint osteoarthritis and, thus, an indication for unicompartmental knee arthroplasty [6, 7]. Because of this effect of tibiofemoral subluxation on the mechanics of the medial knee compartment, tibiofemoral subluxation may increase the incidence of intercondylar notch and tibial eminence impingement. Considering that the medial meniscus (MM) plays a role as a secondary stabilizer during tibial translation [8], with a posterior root tear, the MM is displaced medially and posteriorly [2, 9]. This displacement may induce pathological movements at the knee joint, including a tibiofemoral subluxation.

Biomechanically, the tibiofemoral contact area and contact pressure are higher with the knee in a flexed than extended position [10]. Therefore, radiographs obtained while weight bearing with knee flexion may provide a more reliable assessment of an MMPRT than those taken in an extended position. The assessment of the knee joint in weight bearing is further essential when we consider that tibiofemoral cartilage damage, with associated meniscal extrusion, most likely results from altered weight bearing and load distribution due to a displaced meniscus [11]. Therefore, the pathological loads caused by an MMPRT might result in not only meniscus extrusion but also tibiofemoral subluxation. As such, if tibiofemoral subluxation can be evaluated using radiographic examination, then positive findings on weight-bearing radiography could provide a persuasive and expedient preliminary method for early detection of an MMPRT.

Weight-bearing posterior-anterior (PA) radiograph of the knee in 45° of flexion (known as the Rosenberg view) provides useful insights into the narrowing of the joint space and identification of the intercondylar area and tibial eminence [12]. Moreover, radiographs obtained using this method provide better sensitivity and specificity than conventional standard radiographs to identify intra-articular changes in the joint contact areas of the knee [13]. On the basis of this evidence, our aim in this study was to verify the effectiveness of detecting MMPRTs using the Rosenberg view. We hypothesized that (1) the presence of an MMPRT would affect the distance between the MTE and lateral edge of medial femoral condyle (MFC), as well as the medial joint space (MJS) width and (2) MM extrusion (MME), following an MMPRT, measured using magnetic resonance imaging (MRI), would correlate with the MTE–MFC distance and MJS width.

## Materials and methods

### Study patients

**Fig. 1:**
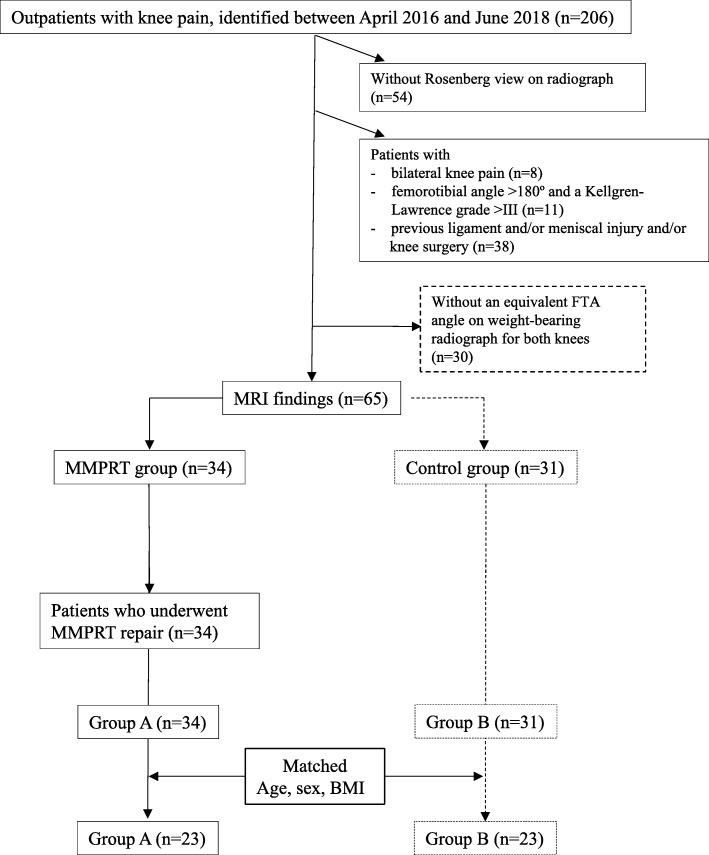
Flow diagram of patient identification and allocation to groups

Patients were eligible for inclusion if they had visited the outpatient department for knee pain (apart from patients requiring total knee arthroplasty) between April 2016 and June 2018 (*n* = 206). Exclusion criteria were as follows: (1) patients without radiographs using the Rosenberg method (*n* = 54); (2) patients with bilateral knee pain (*n* = 8); (3) patients with a femorotibial angle (FTA) > 180° and a Kellgren-Lawrence grade higher than III (*n* = 11); (4) patients with a previous ligament and/or meniscal injury and/or knee surgery (*n* = 38); and (5) patients without an equivalent FTA on weight-bearing radiographs for both knees (*n* = 30). These exclusion criteria were also applied to the contralateral knee that was used for comparison, although the availability of MRI was not required (Fig. 1).

An MRI evaluation was completed for 65 patients, who were classified into two groups: Group A with evidence of an MMPRT (*n* = 34) and Group B with no evidence of an MMPRT (control, *n* = 31). The presence of an MMPRT was based on characteristic MRI findings, including signs of cleft, giraffe neck, ghost, radial tear, and meniscal extrusion within 9 mm of the meniscal attachment [14, 15]. Patients in Group A met the indication for MMPRT pullout repair [16–18]. Groups were then matched for age, sex, and body mass index, with 23 participants included in each of the two groups after matching (Fig. 1). The 23 patients in Group B (control) were diagnosed with other injuries not considered to be a contributing factor to MME [11], including degenerative changes of the MM (*n* = 13), horizontal tears of the lateral meniscus (*n* = 5), a Baker’s cyst (*n* = 3), and impingement of the infrapatellar fat pad, known as Hoffa’s syndrome (*n* = 2) (Fig. 1). The characteristics of the patient groups are summarized in Table 1.

**Table 1:** Demographics and clinical characteristics

	Group A	Group B	*P* value
Number of patients	23	23	
Sex (male/female)	3/20	4/19	0.90
Age (years)	61.5 ± 9.9	59.2 ± 10.1	0.37
Height (m)	1.54 ± 0.16	1.60 ± 0.13	0.32
Body weight (kg)	65.9 ± 16.0	62.8 ± 24.2	0.31
Body mass index (kg/m^2^)	26.3 ± 4.3	26.2 ± 3.9	0.68
Kellgren-Lawrence grade (0/I/II/III/IV)	2/16/10/0/0	2/14/12/0/0	
Femorotibial angle, affected side (°)	177.2 ± 1.5	176.4 ± 1.8	0.23
Femorotibial angle, contralateral side (°)	175.9 ± 1.8	176.2 ± 1.4	0.79
Femorotibial angle of knee flex, affected/contralateral side (°)	170.7 ± 1.9/169 ± 1.3	170.1 ± 1.8/168.2 ± 1.2	0.42/0.48

Data on age, height, body weight, body mass index, and femorotibial angle are presented as mean ± standard deviation

The outcomes of interest included widening of the MTE–MFC distance and the MJS width measured on Rosenberg view radiographs and referenced to the contralateral knee.

### Weight-bearing PA radiographic method (Rosenberg view)

The flexed-knee position used for standing radiographs in our study was the same as the original Rosenberg method [12] except the toes were fixed at 10° of external rotation to control the position of the tibia. The X-ray tube was placed at a distance of 40 inches (101.6 cm) from the knee, centered on the patella in a caudad orientation of 10°. Radiographs of both knees were obtained for comparison. All radiographic images were acquired digitally using a picture archiving and communication system (PACS).

### FTA measurement: standing position and Rosenberg view

The FTA in the standing position was assessed using full-length anterior–posterior radiographs. The femoral shaft line was drawn from the center of the femoral condyle, at the level of the top of the intercondylar notch, to the mid-shaft, at a point 10 cm above the knee. The tibial shaft line was drawn from this mid-shaft point to the midpoint of the talus [19]. The angle subtended by these two lines was recorded as the FTA in standing position (Table 1).

**Fig. 2:**
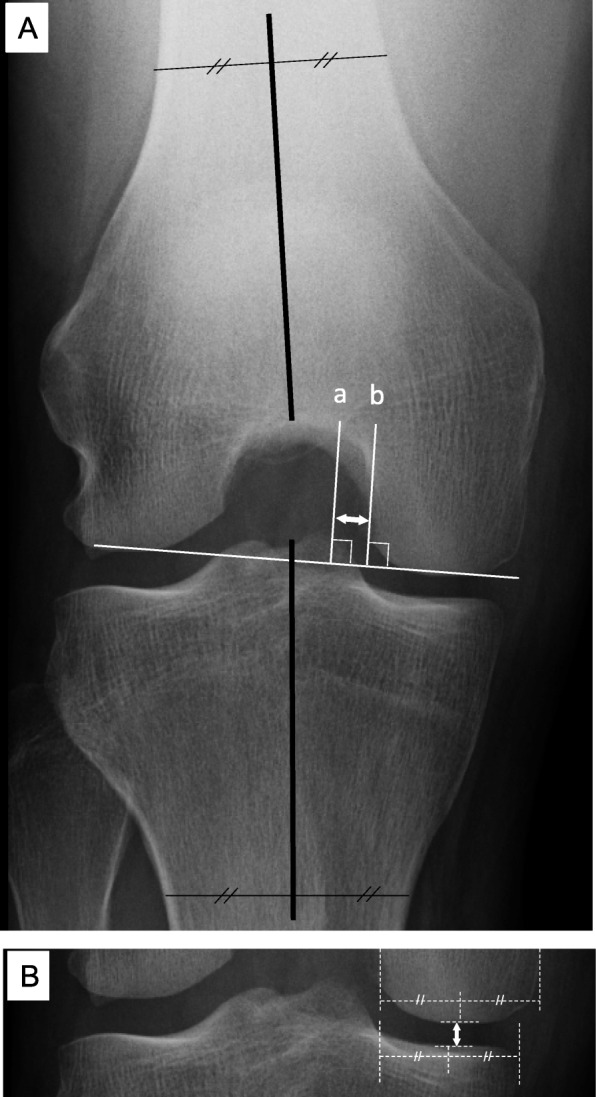
**a** Measurement methods for the distance from the medial tibial eminence (MTE) to the medial femoral condyle (MFC) and the femorotibial angle (FTA) during weight bearing with the knee flexed at 45°. The MTE line (*a*) and MFC line (*b*) are drawn perpendicular to the line tangent to the medial and lateral femoral condyles, along the lateral edge of the MFC. The distance between the MTE and the MFC is then measured (*white double-headed arrow*). **b** Measurement of the medial joint space (MJS) width using the Rosenberg view. The MJS is measured from the center of the MFC to the center of the medial tibial plateau (*white double-headed arrow*). The weight-bearing FTA, with the knee flexed at 45°, is measured (*black lines*) (**a**). A best fit line is drawn through the midpoints of the outer cortex of both the femur and tibia, and the FTA is defined by the angle at their intersection

The weight-bearing FTA, with the knee flexed at 45°, was measured using 30 × 40 cm films. A best fit line was drawn through the midpoints of the outer cortices of both the femur and the tibia, with the angle subtended by their intersection recorded as the FTA in knee flexion [19, 20] (Table 1; Fig. 2a).

### Evaluation of the distance between the MTE and MFC

On the Rosenberg view, the MTE line was drawn perpendicular to the line tangent to the medial and lateral femoral condyles, and the MFC line perpendicular to the line tangent to the medial and lateral femoral condyles, along the lateral edge of the MFC. The distance between the MTE and MFC was measured (Fig. 2a). To compare this distance, the same methods were used for the contralateral knee.

### Evaluation of the MJS

The MJS was measured from the center of the MFC to the center of the medial tibial plateau (Fig. 2b) [21].

### MRI-based measurement of MME

**Fig. 3:**
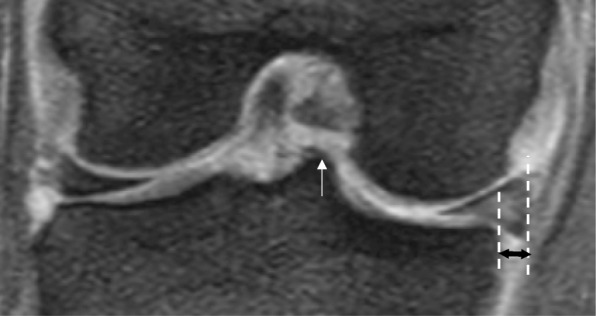
Magnetic resonance image-based measurement of medial meniscus extrusion (MME). Measurement of MME is performed using a bony landmark method. On the coronal plane, the medial tibial eminence (*white arrow*) is used to measure the horizontal distance between the most medial aspect of the tibia and the most medial aspect of the meniscus (*black double-headed arrow*)

MRI evaluation was performed in the supine position, using either an Achieva 1.5 T (Philips, Amsterdam, The Netherlands) or an EXCELART Vantage powered by Atlas 1.5 T (Toshiba Medical Systems, Otawara, Japan), with a knee coil. Measurement of MME was performed using a bony landmark method, which is considered more reproducible than a coronal slice method [22]. A coronal plane through the MTE was used to measure the horizontal distance between the most medial aspect of the tibia and the most medial aspect of the meniscus on this image (Fig. 3).

**Fig. 4:**
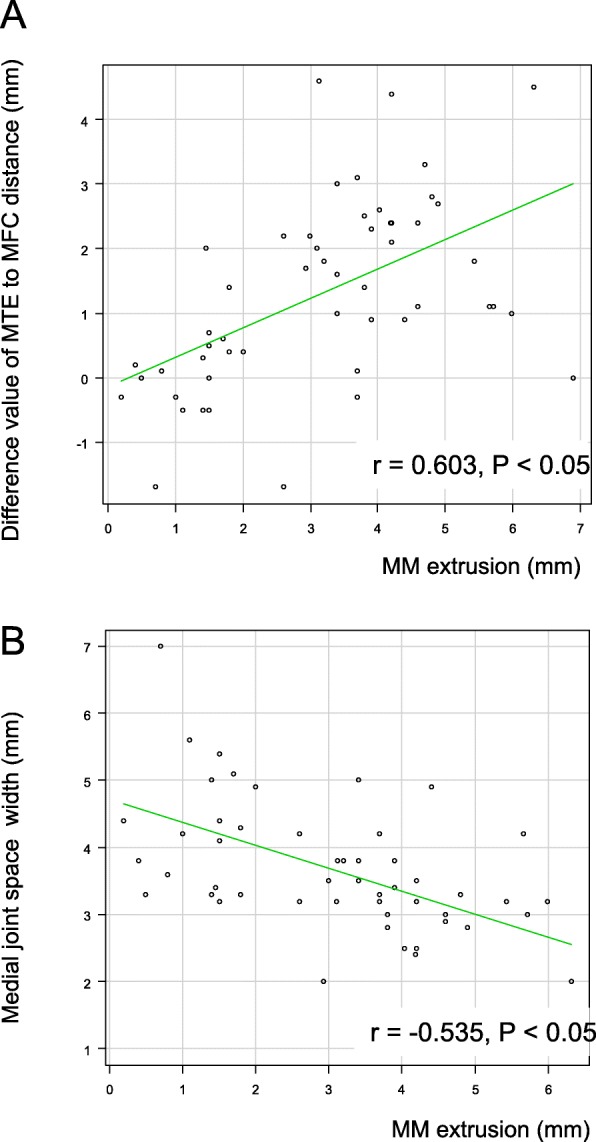
**a** Correlation of medial meniscus extrusion (MME) with the distance between the medial tibial eminence (MTE) and medial femoral condyle (MFC). The correlation coefficient between MME and MTE–MFC distance is r = 0.603, *P* < 0.05. **b** Correlation between MME and medial joint space (MJS). The correlation coefficient between MME and MJS is r = − 0.535, *P* < 0.05

### Statistical analysis

Descriptive data are presented as the mean ± standard deviation. Differences in age, height, body weight, body mass index, and FTA between Group A and Group B were evaluated using the Mann–Whitney U-test. Between-group differences for sex-related data were evaluated using the Fisher’s exact test. We compared the MTE–MFC distance and MJS width between the affected and unaffected knees and evaluated the association of the extent of MME with the MTE–MFC distance and MJS width in knees with and without a MMPRT using Spearman’s rank correlation. All analyses were performed using the EZR-WIN software (Saitama Medical Center, Saitama, Japan), which is a graphical user interface for R (The R Foundation for Statistical Computing) [23]. Statistical significance was set at *P* < 0.05 a priori.

### Intra- and interobserver repeatability

Two orthopedic surgeons independently assessed the Rosenberg radiographs and MRIs in a blinded manner. Each observer performed each evaluation twice, at least 2 weeks apart. The averages of these measurements were used in the calculation of the intraclass correlation coefficient (ICC) for intra- and interobserver repeatability for the measurement of the MTE–MFC distance, MJS width, and MME.

## Results

The demographics and clinical characteristics were not significantly different between the two groups (Table 1). The extent of MME was greater in Group A than in Group B (4.1 ± 1.1 mm versus 1.8 ± 1.5 mm; *P* < 0.05). The MTE–MFC distance was significantly greater in Group A than in Group B (7.7 ± 1.7 mm versus 6.0 ± 1.2 mm; *P* < 0.05). The MJS width was significantly smaller in Group A than in Group B (3.2 ± 0.8 mm versus 4.5 ± 0.7 mm; *P* < 0.05). Compared to the contralateral knee, the affected knee had a greater MTE–MFC distance and smaller MJS width (2.2 ± 1.0 mm versus − 0.1 ± 0.7 mm and − 1.3 ± 0.7 mm versus − 0.1 ± 0.6 mm, respectively; *P* < 0.05: Table 2).

**Table 2:** Evaluation of medial meniscus extrusion using MRI and comparison of MTE–MFC distance and medial joint space width using the Rosenberg view in the two groups

	Group A	Group B	*P* value
Medial meniscus extrusion (range)	4.1 ± 1.1 (3.0–5.7)	1.8 ± 1.5 (0.2–2.0)	< 0.05
MTE–MFC distance (range)	7.7 ± 1.7 (4.9–12.9)	6.0 ± 1.2 (2.9–7.4)	< 0.05
Difference between affected and contralateral knee (range)	2.2 ± 1.0 (0.9–4.6)	− 0.1 ± 0.7 (− 1.7–1.4)	< 0.05
Medial joint space width (range)	3.2 ± 0.8 (2.4–6.5)	4.5 ± 0.7 (3.8–7.0)	< 0.05
Difference between affected and contralateral knee (range)	−1.3 ± 0.7 (− 0.4–-2.8)	−0.1 ± 0.6 (− 1.7–0.6)	< 0.05

Data are presented as a mean ± standard deviation. Statistical differences were evaluated using the Student’s *t* test

*MRI* magnetic resonance imaging, *MTE* medial tibial eminence, *MFC* medial femoral condyle

A good correlation was observed between the MME measurement on MRI and the MTE–MFC distance (quantified as the difference between the affected and contralateral knees) on radiographic examination (r = 0.603, *P* < 0.05; Fig. 4a). The MME measurement and the MJS width were also correlated (r = − 0.535, *P* < 0.05; Fig. 4b).

Measurement of the MTE–MFC distance was consistent, with ICC values of 0.928–0.962 for intraobserver repeatability and 0.984–0.991 for interobserver repeatability. Similarly, for the MJS width measurement, the ICCs ranged between 0.936 and 0.958 for intraobserver repeatability and 0.938 and 0.978 for interobserver repeatability. For the MME measurement, the ICCs for intraobserver repeatability and interobserver repeatability ranged between 0.899 and 0.912 and between 0.902 and 0.925, respectively.

## Discussion

This study evaluated whether the presence of an MMPRT would affect the MTE–MFC distance and MJS width, measured on the Rosenberg view. We also evaluated whether the MME measured on MRI in the supine position correlated with the MTE–MFC distance and the MJS width. The most important finding of our study was the association between the presence of an MMPRT and a greater MTE–MFC distance and smaller MJS width, with these intra-articular changes correlating to the MME measured on MRI.

**Fig. 5:**
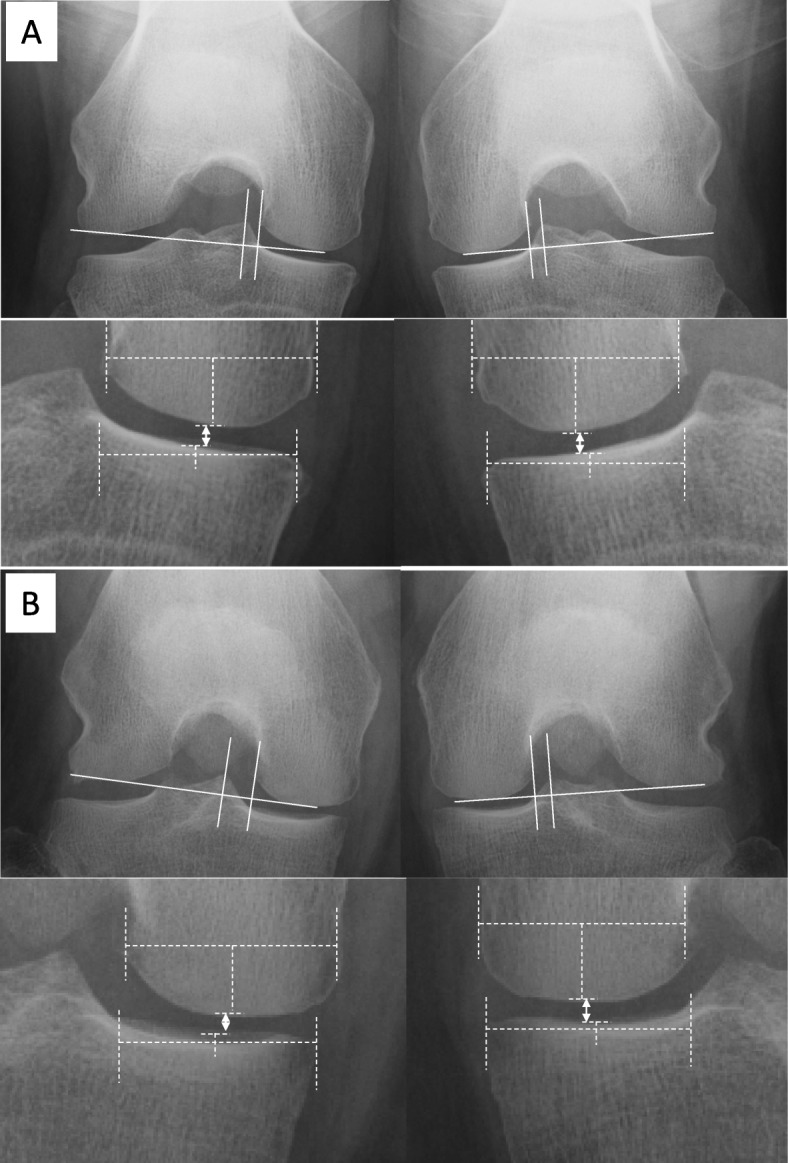
Representative posterior-anterior weight-bearing radiographs (Rosenberg view). **a** A 62-year-old woman was diagnosed with degenerative changes in the medial meniscus. The distance between medial tibial eminence to the medial femoral condyle (MTE–MFC distance): *right*, 3.3 mm; *left*, 3.1 mm. Medial joint space (MJS) width: *right*, 4.3 mm; *left*, 4.3 mm. **b** A 63-year-old woman was diagnosed with a right medial meniscus posterior root tear. MTE–MFC distance: *right*, 6.8 mm; *left*, 4.2 mm. MJS width: *right*, 2.8 mm; *left*, 4.3 mm. The *white double-headed arrows* indicate the MJS width

Plain radiographic examination of the knee under loading conditions is recommended when examining patients with meniscal tears [24]. Meniscal extrusion after an MMPRT often leads to radiographic changes, such as MJS narrowing and varus deformity of the knee [25]. We demonstrated that these intra-articular changes, caused by MMPRT, are measurable on the radiographic Rosenberg view (Fig. 5).

The evidence relating tibiofemoral subluxation to MMPRT is weak overall; only one cadaveric study regarding the kinematics of the tibia associated with MMPRT reported a tibiofemoral subluxation of about 2 mm over the arc of 30–60° of knee flexion in non-weight bearing [1]. In our study, we evaluated the MTE–MFC distance on the Rosenberg view, observing a greater MTE–MFC distance (of 2.2 mm on average) associated with an MMPRT in the affected knee compared to the unaffected knee. The 2.2-mm increase in the MTE–MFC distance was comparable to the 2-mm tibiofemoral subluxation shown in a previous cadaveric study [1]. In addition, MME is associated with a loss of medial compartment cartilage volume [26] and MJS narrowing [27]. In general, the intact middle horn of the MM buttresses against the medial portion of the MFC [1]. Loss of this buttress, resulting from tibiofemoral dysfunction due to MM dysfunction, may result in failure of secondary stabilizers crucial for tibial stability and, thus, leads to tibiofemoral subluxation [8].

Although MRI examination is indispensable for the diagnosis of MMPRT, considering the time required for imaging and the high cost, MRI should only be performed in the presence of certain clinical evidence to support its use. On the other hand, it is important to not miss the diagnosis of an MMPRT because of the associated risk for progression of degenerative changes over a short period [28]. Furthermore, if surgery is performed at a later stage, improvements in the MME cannot be expected [29]. Therefore, early detection and treatment of an MMPRT can provide the best patient-related outcomes.

In our protocol, we used the Rosenberg view with weight-bearing PA radiographs, with the weight equally distributed on the two limbs and the foot in a position of 10° of external rotation. However, as the MTE–MFC distance may be influenced by the rotation of the tibia, some caution is required in the interpretation of our results. We note that the FTA measured in a weight-bearing position with the knee in 45° of flexion was equal on both sides, and that the positional relationship between the fibula head and the lateral aspect of the tibia was also equal on both sides; these factors are important indicators of tibial rotation. Despite the standardization of tibial rotation for measurement on the Rosenberg view, the MTE–MFC distance may be influenced by muscle contraction. The internal rotation of the tibia is influenced by the properties and activities of various muscles, including the popliteal muscle and the semitendinosus, semimembranosus, sartorius, and gracilis muscles. However, in a study evaluating the rotation of the tibia using upright weight-bearing computed tomography (CT) imaging, internal rotation of the tibiofemoral joint did not increase between 30° and 60° of knee flexion [25]. Therefore, considering the Rosenberg view is obtained with the knee in 45° of flexion, we infer that the internal rotation of the tibia during imaging was not influenced by the state of muscle contraction. Therefore, the measurement of the MTE–MFC distance using the Rosenberg view can be considered to be a useful and reproducible method for the preliminary diagnosis of an MMPRT.

There were several limitations in this study. First, patients were retrospectively assessed and the number of cases studied was small. Second, the knee joints of middle-aged and older patients commonly show some injury to the MM such that the MM on the unaffected knee, used as control reference, was rarely normal. Therefore, to match the conditions as much as possible, we selected patients with degeneration changes without meniscal tears that would influence MME to form the control group. Third, we evaluated the positional relationship between the tibia and the femur using two-dimensional radiographs. In this study, however, we did not evaluate the rotation of the tibiofemoral joint, so future studies should evaluate the rotation using weight-bearing CT or MRI. Considering the function of the meniscus and the rotation of the femur and the tibia in patients with MMPRT, three-dimensional reconstruction of the MM using dynamic MRI may be useful to understand the pathological kinematics of the tibiofemoral joint and MME increase after MMPRT.

## Conclusions

This study demonstrates that the presence of an MMPRT increases the MTE–MFC distance and decreases the MJS width, measured on the Rosenberg view. In addition, MME assessed by MRI in the knee with an MMPRT correlated with the MTE–MFC distance and MJS width on the weight-bearing radiographs. Therefore, assessment of the MTE–MFC distance and MJS width using the Rosenberg view may be a useful preliminary method for the diagnosis of an MMPRT to be confirmed by MRI.
